# Supplementary material for: Drug Utilization and Medication Adherence: A Data-Driven Analysis of Drugs with Different Routes of Administration Applied in Atopic Dermatitis
Source: Pharmaceutics. 2025 Oct 1;17(10):1279. doi: 10.3390/pharmaceutics17101279 (PMC12567112; doi:10.3390/pharmaceutics17101279)
Supplement: Supplementary file 1 [file pharmaceutics-17-01279-s001.zip › pharmaceutics-3814933-supplementary.pdf]

Supplementary Material

Drug Utilization and Medication Adherence: A Data-Driven Analysis of Drugs with Different Routes of Administration Applied in Atopic Dermatitis

Legend

Figure S1. Schematic presentation of the study design and cohort selection.

Table S1. Overview of AD pharmacological treatments.

Table S2. Comparison of drug characteristics for tacrolimus ointment and dupilumab injection treatments for moderate/severe AD.

Figure S1. Schematic presentation of the study design and cohort selection.

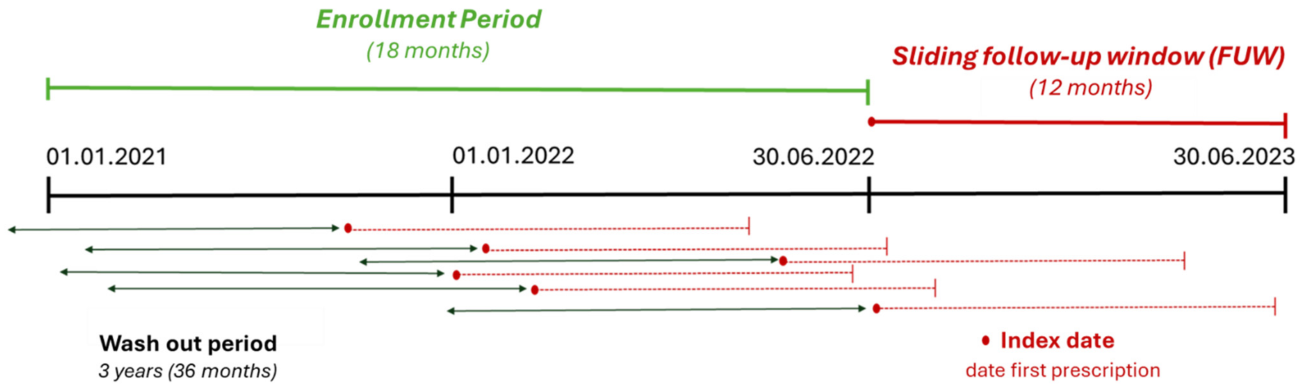

Table S1. Overview of AD pharmacological treatments.

ATC code	Active Substance	Trade Name	Posology	Drug speciality code	Year of Marketing Authorisation	Therapeutic Indications	Reimbursability*	Ex Factory Price	Public Price (VAT included)
**D07 Corticosteroids, dermatological preparations**									
**D07AC14**	**Methylprednisone aceponate**	**Advantan**	0,1% cream 20g	028159010	12 Dec 1999	eczema vulgaris; allergic and irritative contact eczema; dyshidrotic eczema; eczema of children, scalp dermatoses of inflammatory character, nummular eczema, eczema vulgaris	**C**	/	€ 7.90
			0,1% hydrophobic cream 20g	028159022			**C**	/	€ 7.90
			0,1% skin solution 20g	028159046			**C**	/	€ 7.90
			0,1% ointment 20g	028159034			**C**	/	€ 7.90
			0,1% 50g cream	028159073			**A**	€ 5.89	€ 9.73
			1 pack 50g emulsion	028159061			**C**	/	€ 12.00
**D11 Other dermatological preparations**									
**D11AH02**	**Pimecrolimus**	**Elidel**	10 mg/g (1%) of 15 g	036006017	01 Dec 2004	mild to moderate atopic dermatitis (2 years old and older)	**C**	/	€ 28.30
			10 mg/g (1%) of 30 g	036006029			**H**	€ 23.84	€ 39.34
**D11AH05**	**Dupilumab**	**Dupixent**	300 mg/2ml	045676069	26 Sep 2017	moderate to severe atopic dermatitis in adults and adolescents aged 12 years and older eligible for systemic therapy, severe atopic dermatitis in children aged 6 to 11 years, eligible for systemic therapy, asthma, chronic rhinosinusitis with nasal polyposis	**H**	€ 1,155.20	€ 1,906.54
			200 mg/1,14ml	045676107			**H**	€ 1,155.20	€ 1,906.54
			200mg/1,14ml	045676145			**H**	€ 1,155	€ 1,906.54
			300mg/2ml	045676184			**H**	€ 1,155	€ 1,906.54
**D11AH07**	**Tralokinumab**	**Adtralza**	150mg	049573025	17 Jun 2021	atopic dermatitis	**H**	€ 1,280	€ 2,112.51
**D11AH08**	**Abrocitinib**	**Cibinqo**	50mg	049826011	09 Dec 2021	moderate to severe atopic dermatitis in adult patients	**C (nn)**	/	/
			100mg	049826062			**C (nn)**	/	/

ATC code	Active Substance	Trade Name	Posology	Drug speciality code	Year of Marketing Authorisation	Therapeutic Indications	Reimbursability*	Ex Factory Price	Public Price (VAT included)
			200mg	049826112			**C (nn)**	/	/
**H02 Systemic corticosteroids**									
**H02AB02**	**Dexamethasone**	**Soldesam**	4mg/ml 3 vials	019499019	21 Dec 1961	anti-inflammatory corticotherapy, degenerative and post-traumatic arthritis, inflammatory arthritis, chronic developmental polyarthritis, ankylosing spondylarthritis, asthmatic accesses, cerebral edema, cerebral neoplasms (as adjuvant), emergency and shock states: edema of the glottis, post-transfusion reactions, anaphylaxis; haemorrhagic, surgical, septic, cardiogenic, burn trauma, asthmatic states, allergic dermatitis and dermatoses, atopic dermatitis, contact dermatitis, seborrheic dermatitis, intertrigo	**A**	€ 1.69	€ 2.79
			0,2% gtt/10ml	019499072			**A**	€ 2.55	€ 4.21
			8mg/2ml 3 vials	019499084			**A**	€ 2.90	€ 4.79
**H02AB04**	**Methylprednisolone**	**Medrol**	2mg/30 tabs	014159065	31 May 2005	endocrine disorders, rheumatologic diseases, collagenopathies, dermatologic diseases, allergic states, ophthalmic diseases, hematologic diseases, neoplastic diseases, edematous states, respiratory diseases, gastrointestinal diseases	**C**	4.87	€ 8.00
			4mg/30 tabs	042861017			**C**	/	€ 8.95
			16mg/10 tabs	014159040			**A**	€ 3.75	€ 6.20
**H02AB07**	**Prednisone**	**Deltacortene**	5mg/10 tabs	010089011	01 Jun 2010	rheumatologic diseases, systemic lupus erythematosus, dermatomyositis, periarthritis, rheumatic carditis, severe or debilitating allergic conditions, sarcoidosis, hematologic conditions, ulcerative colitis, palliative of adult leukaemia	**A**	€ 0.99	€ 1.62
			5mg/20 tabs	010089047			**A**	€ 1.97	€ 3.25
			15mg/10 tabs	010089035			**A**	€ 3.27	€ 5.82

ATC code	Active Substance	Trade Name	Posology	Drug speciality code	Year of Marketing Authorisation	Therapeutic Indications	Reimbursability*	Ex Factory Price	Public Price (VAT included)
						and lymphoma and acute childhood leukemia			
**L04 Immunosuppressants**									
**L04AA44**	**Upadacitinib**	**Rinvoq**	30 mg	048399063	16 Dec 2019	moderate to severe atopic dermatitis in adults and adolescents aged 12 years and older, eligible for systemic therapy, rheumatoid arthritis, psoriatic arthritis, and ankylosing spondylitis	**C (nn)**	/	/
**L04AA37**	**Baricitinb**	**Olumiant**	2mg/28tabs	045260027	13 Feb 2017	moderate to severe atopic dermatitis in adult patients who are candidates for systemic therapy, moderate to severe active phase rheumatoid arthritis in adult patients	**H**	€ 660.21	€ 1,089.61
			4mg/28tabs	045260104			**H**	€ 660.21	€ 1,089.61
			4mg/84tabs	045260142			**H**	€ 1,980.64	€ 3,268.85
**L04AD01**	**Ciclosporin A**	**Ciqorin**	100 mg/30 caps	042787299	/	organ transplantation, bone marrow transplantation, endogenous uveitis, nephrotic syndrome, rheumatoid arthritis, psoriasis, atopic dermatitis	**A**	€ 29.52	€ 55.32
			10mg/50caps	042787251			**A**	€ 5.42	€ 10.17
			25mg/ 50caps	042787263			**A**	€ 13.55	€ 25.41
			50mg/50caps	042787275			**A**	€ 26.27	€ 49.28
**L04AD02**	**Tacrolimus**	**Protopic**	0,03% 30g	035575012	10 Aug 2012	moderate to severe atopic dermatitis in adults (from 16 years of age), moderate to severe atopic dermatitis in children (from 2 years of age), kidney transplant rejection, liver transplant rejection.	**A**	€ 20.18	€ 33.31
			0,03% 10g	035575051			**A**	€ 6.73	€ 11.11
			0,1% 10g	035575063			**A**	€ 7.56	€ 12.48
			0,1% 30g	035575036			**A**	€ 22.70	€ 37.46
**L04AX01**	**Azatioprina**	**Aspen**	50mg/50tabs	020957039	30 Oct 1972	immunosuppressive and antimetabolite, chronic inflammatory bowel disease (Crohn's disease, ulcerative colitis), severe rheumatoid arthritis, systemic lupus erythematosus, dermatomyositis and polymyositis, autoimmune	**A**	€ 8.93	€ 14.73

ATC code	Active Substance	Trade Name	Posology	Drug speciality code	Year of Marketing Authorisation	Therapeutic Indications	Reimbursability*	Ex Factory Price	Public Price (VAT included)
						hemolytic anemia, refractory chronic idiopathic thrombocytopenic purpura			
L04AX03	Methotrexate	Reumaflex	10mg/0,2 ml	039153123	29 Dec 2009	active rheumatoid arthritis in adult patients, polyarthritic forms of severe juvenile idiopathic arthritis in an active phase, severe psoriasis, Crohn's disease	A	€ 17.85	€ 29.46
			12,5mg/0,25ml	039153628			A	€ 18.20	€ 34.14
			15mg/0,30ml	039153224			A	€ 26.07	€ 43.02
			17,5mg/0,35ml	039153679			A	€ 25.09	€ 47.05
			20mg/0,40ml	039153325			A	€ 34.48	€ 56.90
			25mg/0,50ml	039153426			A	€ 42.34	€ 69.88
**R03 Drugs for chronic obstructive pulmonary disease**									
	Fluticasone propionate	Flixoderm	0,05mg/g ointment	029014038	19 Mar 1998	Sensitive dermatoses (in adults and children one year of age and older) such as: eczema, prurigo nodularis, psoriasis, neurodermatosis, including lichen simplex, lichen planus, seborrheic dermatitis, allergic contact reactions, discoid lupus erythematosus, generalized erythroderma in association with systemic steroid therapy, insect bites, miliaria rubra	A	€ 3.74	€ 6.17
			0,5mg/g cream	029014014			A	€ 3.35	€ 5.53
**R06 Antihistamines for systemic use**									
R06AE06	Oxatomide	Tinset	30mg/30tabs	025293010	01 Feb 2012	prevention and basic treatment of allergic conditions, mainly in cases of rhinitis, extrinsic asthma, follicular conjunctivitis, chronic urticaria, atopic dermatitis, and food allergies	A	€ 3.26	€ 5.38
			2,5% 30ml gtt	025293034			A	€ 3.01	€ 4.98
R06AE07	Cetirizine	Zirtec	10mg/20tabs	026894016	26 May 2008	treatment of nasal and ocular symptoms of seasonal and perennial allergic rhinitis, symptomatic treatment of chronic idiopathic urticaria	A	€ 5.21	€ 8.60
			10mg/ml gtt	026894028			A	€ 6.60	€ 10.90

ATC code	Active Substance	Trade Name	Posology	Drug speciality code	Year of Marketing Authorisation	Therapeutic Indications	Reimbursability*	Ex Factory Price	Public Price (VAT included)
**R06AE09**	**Levocetirizine**	**Xyzal**	5mg/20tabs	035666080	27 May 2003	symptomatic treatment of allergic rhinitis (including persistent allergic rhinitis), chronic idiopathic urticaria in adults and children 6 years of age and older	**A**	€ 3.45	€ 5.68
			5mg/ml gtt	035666205			**A**	€ 5.75	€ 9.48
**R06AX22**	**Ebastine**	**Kestine**	20mg/20tabs	034930026	01 Jun 2003	allergic rhinitis (seasonal or perennial) associated or not with allergic conjunctivitis, urticaria (indication authorized only for Kestine 10 mg)	**C**	/	€ 21.90
			10mg/30tabs	034930014	01 Dec 2001		**A**	€ 6.03	€ 9.96
			10mg/30 lyophilized doses	034930141			**A**	€ 6.03	€ 9.96
**R06AX28**	**Rupatadine Fumarate**	**Pafinur**	10mg/30tabs	037888068	12 May 2008	allergic rhinitis (including persistent allergic rhinitis) in children aged 2-11 years, urticaria in children aged 2-11 years	**A**	€ 5.75	€ 9.76
			1mg/ml oral solution 120ml	037888094			**A**	€ 5.38	€ 8.87

Abbreviations: A: essential drugs and those for chronic diseases, fully reimbursed by the Italian National Health Service (NHS); H: drugs for hospital use only that can be used only in hospitals and/or provided in the context of hospitals and assimilated facilities; C (nn): drugs with a European marketing authorisation (MA) but not yet negotiated by the Italian medicine agency for reimbursability.

Supplementary Table S2. Comparison of drug characteristics for tacrolimus ointment and dupilumab injection treatments for moderate/severe AD.

Drug characteristics	Tacrolimus Ointment	Dupilumab Injection
**Formulation**	Topical immunomodulatory ointment	Subcutaneous monoclonal antibody (anti-IL-4Rα)
**Route of administration**	Topical application	Subcutaneous injection
**Approved AD severity**	Moderate-to-severe AD (when corticosteroids are ineffective or not tolerated)	Moderate-to-severe AD not adequately controlled with topical therapies
**Line of treatment**	First or second-line (depending on severity and corticosteroid tolerance)	Third-line or systemic option after failure of topicals
**Age indications**	≥ 2 years	≥ 6 months (as per latest updates)
**Frequency of administration**	Twice daily (initially), can be reduced after improvement	Every 2 weeks (adults), weekly for children (weight-based)
**Prescriber setting**	Hospital or specialist centres, (dermatologists, allergologists, paediatricians)	Hospital or specialist centres, (dermatologists, immunologists, pulmonologists, allergologists, paediatricians)
**Monitoring requirements**	Not required, although long-term use caution advised	Monitor for systemic reactions, eosinophilia, conjunctivitis, hypersensitivity
**Self-administration feasibility**	Requires careful application, may be inconvenient for some patients	Requires initial training, but highly feasible for long-term self-injection

Sources: Summaries of product characteristics for tacrolimus ointment and dupilumab injection approved in Italy [1,2] and Italian guidelines from SIDeMaST, Italian Society of Medical, Surgical, Aesthetic Dermatology and Sexually Transmitted Diseases [3].

References

1. Protopic. Summary of Product Characteristics.Astellas Ireland Co. Ltd. & LEO Laboratories Ltd; 2006. Available on: https://api.aifa.gov.it/aifa-bdf-eif-be/1.0.0/organizzazione/819/farmaci/35575/stampati?ts=RCP. Accessed Sep 8, 2025.
2. Dupixent. Summary of product characteristics. Sanofi Winthrop Industrie; 2022.Available on: https://www.ema.europa.eu/en/documents/product-information/dupixent-epar-product-information_hr.pdf. Accessed Sep8, 2025.
3. AA VV. Linee Guida e Raccomandazioni SIDeMaST. Pisa: Pacini Editore; 2011.Available on: https://www.pacinimedicina.it/prodotto/linee-guida-e-raccomandazioni-sidemast/. Accessed Sep 8, 2025.
